# Development of Erf-Mediated Craniosynostosis and Pharmacological Amelioration

**DOI:** 10.3390/ijms24097961

**Published:** 2023-04-27

**Authors:** Angeliki Vogiatzi, Kleoniki Keklikoglou, Konstantinos Makris, Dionysia Stamatia Argyrou, Athanasios Zacharopoulos, Varvara Sotiropoulou, Nikolaos Parthenios, Angelos Gkikas, Maria Kokkori, Melodie S. W. Richardson, Aimée L. Fenwick, Sofia Archontidi, Christos Arvanitidis, Jeremy Robertson, John Parthenios, Giannis Zacharakis, Stephen R. F. Twigg, Andrew O. M. Wilkie, George Mavrothalassitis

**Affiliations:** 1 Medical School, University of Crete, 71003 Heraklion, Crete, Greece; 2 IMBB, FORTH, 71003 Heraklion, Crete, Greece; 3 Institute of Marine Biology, Biotechnology and Aquaculture (IMBBC), Hellenic Centre for Marine Research (HCMR), P.O. Box 2214, 71003 Heraklion, Crete, Greece; 4 Biology Department, University of Crete, 71003 Heraklion, Crete, Greece; 5 IESL, FORTH, 71003 Heraklion, Crete, Greece; 6 ICE-HT, FORTH, 26504 Patras, Peloponissos, Greece; 7 Department of Chemistry, University of Oxford, Chemistry Research Laboratory, Mansfield Road, Oxford OX1 3TA, UK; 8 MRC Weatherall Institute of Molecular Medicine, University of Oxford, Oxford OX3 9DS, UK; 9 LifeWatch ERIC, Sector II-II, Plaza de España, 41071 Seville, Spain

**Keywords:** Ets2 repressor factor, craniosynostosis, pharmacological modulation

## Abstract

ETS2 repressor factor (*ERF*) insufficiency causes craniosynostosis (CRS4) in humans and mice. ERF is an ETS domain transcriptional repressor regulated by Erk1/2 phosphorylation via nucleo-cytoplasmic shuttling. Here, we analyze the onset and development of the craniosynostosis phenotype in an Erf-insufficient mouse model and evaluate the potential of the residual Erf activity augmented by pharmacological compounds to ameliorate the disease. Erf insufficiency appears to cause an initially compromised frontal bone formation and subsequent multisuture synostosis, reflecting distinct roles of Erf on the cells that give rise to skull and facial bones. We treated animals with Mek1/2 and nuclear export inhibitors, U0126 and KPT-330, respectively, to increase Erf activity by two independent pathways. We implemented both a low dosage locally over the calvaria and a systemic drug administration scheme to evaluate the possible indirect effects from other systems and minimize toxicity. The treatment of mice with either the inhibitors or the administration scheme alleviated the synostosis phenotype with minimal adverse effects. Our data suggest that the ERF level is an important regulator of cranial bone development and that pharmacological modulation of its activity may represent a valid intervention approach both in CRS4 and in other syndromic forms of craniosynostosis mediated by the FGFR-RAS-ERK-ERF pathway.

## 1. Introduction

Cranial sutures are centers of calvarial bone apposition and growth and have a crucial role in the coordination of infant skull and brain development [1,2]. When one or more of the cranial sutures close prematurely (a condition termed craniosynostosis), the skull cannot conform with the expanding brain, and craniofacial deformities and complications arise that, unless treated, can affect vision, hearing and breathing ability, even causing death. Surgical intervention in early infancy (for example, cranial vault remodeling and distraction osteogenesis) is the only therapeutic approach currently available [3,4]. Craniosynostosis is caused by both environmental and genetic factors, with the latter usually producing a syndromic form of the disease. Variants in a number of genes have been shown to cause craniosynostosis, notably, activating mutations affecting the fibroblast growth factor (FGF) and ERK pathways [5,6,7,8,9].

We, and others, have previously reported that ERF haploinsufficiency causes premature suture closure in humans. This disorder, known as ERF-related craniosynostosis (CRS4; OMIM 61188), exhibits a wide expressivity range in humans from multisuture synostosis to apparent nonpenetrance. Children affected by CRS4 at infancy are frequently considered initially as FGFR syndromic cases, while in other CRS4 cases, there is a delayed onset that, if not diagnosed and treated, can lead to severe complications [10,11].

ERF is a ubiquitously expressed ETS domain transcriptional repressor and a downstream effector of the receptor tyrosine kinase (RTK)–ERK pathway. Regulation of ERF is by direct Erk1/2 multisite phosphorylation leading to nuclear export and inactivity [12]. We have shown that increased nuclear localization of Erf can suppress cellular proliferation and mitogenic activation and can antagonize ETS domain transactivating factors [12,13,14,15]. In contrast, its absence can affect the differentiation of multiple cell types and can cause malignant transformation [16,17,18,19,20,21]. Recently, we showed that Erf is required for the efficient commitment of suture-derived mesenchymal stem/progenitor cells toward the osteogenic lineage via the retinoic acid (RA) pathway [22]. As RA acts as a morphogen and is reported to have a role in bone development, precise control of ERF nuclear levels and transcriptional effects appear to be important for multiple cellular fates.

Our mouse model of Erf-insufficiency closely resembles the human craniosynostosis disorder [11]. In contrast to humans, however, mice heterozygous for *Erf* (*Erf*^+/−^) are phenotypically normal. Mice carrying a hypomorphic allele of *Erf* (*Erf^loxP^* allele), producing roughly 60% Erf protein levels compared to the wild-type allele, combined with a knock-out allele, *Erf^loxP/−^* mice, exhibit a clear synostosis phenotype with multiple suture closures and facial dysmorphism. *Erf^loxP/−^* mice have no other obvious skeletal defects beyond postnatal craniosynostosis and a mild embryonic delay in the ossification of calvarial bones [11]. However, the disease progression during development and penetrance in the mouse model has not been studied in detail. Furthermore, the variable phenotype found in humans carrying heterozygous loss-of-function *ERF* mutations, as well as the absence of any phenotype in *Erf^+/−^* (as opposed to the *Erf^loxP/−^*) mice, indicate that a threshold level of Erf required for normal calvarium development may be the cause of the variable disease expressivity. This may indicate a homeostatic quantitative regulation of cells involved in suture development, as suggested also by various gain-of-function *FGFR* mutations exhibiting a range of synostosis phenotypes [23]. If this threshold hypothesis is valid, it should provide an opportunity to ameliorate the disease phenotype by augmenting the function of residual Erf protein.

In the present study, we examine the progress and penetrance of craniosynostosis and address the hypothesis of a threshold requirement by utilizing our *Erf*-insufficient mouse model to explore possible pharmacological mitigations. Delayed frontal bone development and posterior frontal (PF) suture formation, along with the first signs of synostosis, are evident in postnatal day 15 (P15) mice. The disease progresses during development, and at P65, the multisuture craniosynostosis phenotype is the main characteristic of *Erf^loxP/−^* mice. The treatment of animals with compounds that can enhance Erf nuclear accumulation and repressor function, and analysis of skulls by microcomputed tomography (microCT), support the validity of the functional augmentation hypothesis and suggest that Erf can be a viable pharmacological target in craniosynostosis.

## 2. Results

### 2.1. Erf Insufficiency in Mice Is Associated with Both Craniosynostosis and a Defect in the Development of Frontal Bones

**Figure 1:**
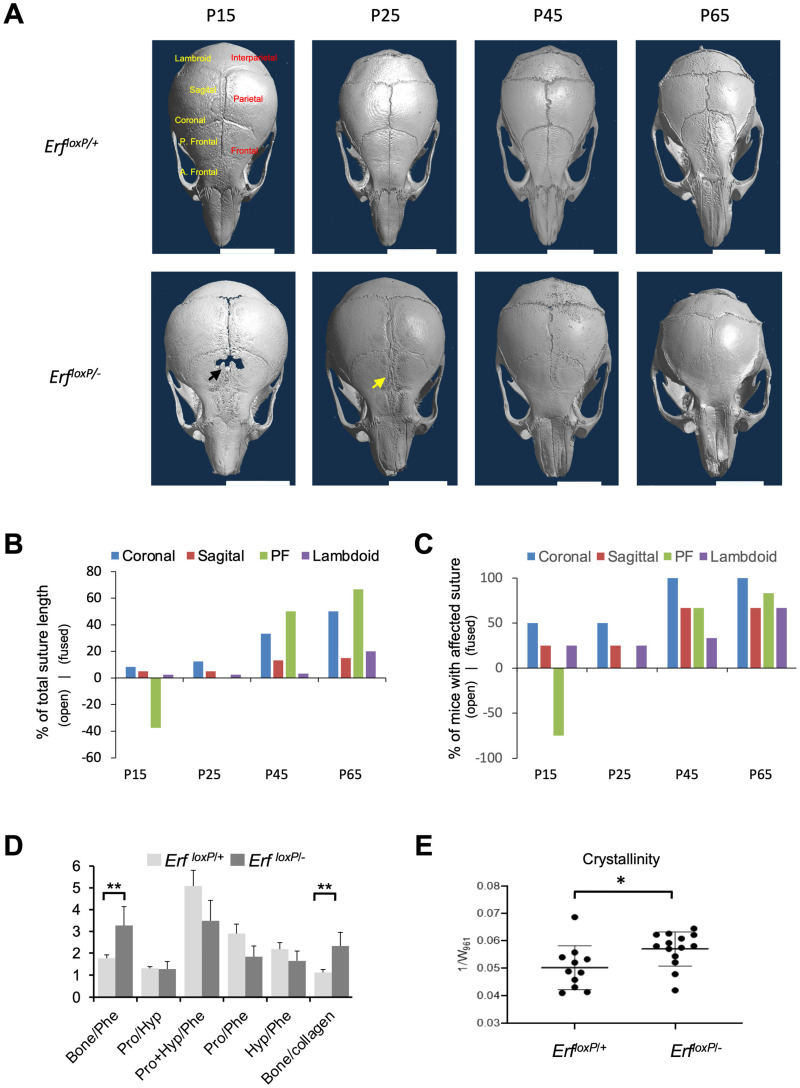
The development and penetrance of craniosynostosis phenotype in *Erf^loxp/−^* mouse model. (**A**) Representative microCT images of mouse skulls at the indicated postnatal time points. The black arrow indicates frontal bone defect. The yellow arrow indicates the deformity of the frontal suture. Cranial sutures in top left panel are named in yellow (P., posterior, A., anterior) and bones in red letters. White scale bars, 5 mm. (**B**) Graph showing the percentage of the total length of each suture that is either closed (positive *y*-axis) or open in comparison to control (negative *y*-axis) considering all the animals at the indicated developmental stages. (**C**) The percentage of mice exhibiting a defect in the indicated suture for each developmental group is shown. Positive values in the *y*-axis are used when the defect is a premature closure of the suture and negative values when the defect involves a widely open suture with a gap, as shown by the black arrow in (**A**). The PF suture at P25, although displaying deformities, is neither closed nor more open compared to the control state. Each measurement in (**B**,**C**) is based on four male mice except for the P45 group, which includes three male mice. The P65 group includes the 6 mice from Figure 5A below. (**D**,**E**) The ratio of phosphates (bone), collagen (Phe), proline (Pro) and hydroxyproline (Hyp) shown in (**D**) and crystallinity shown in (**E**) was evaluated by Raman spectroscopy of coronal sutures from P65 Erf-competent (*Erf^loxP/+^*) and Erf-insufficient (*Erf^loxP/^*^−^) mice. Significant differences were observed in the mineralization of the suture but not the components of the suture. Spectra recorded from the area of the suture with the minimal I961/I1005 ratio and the collagen content relative to mineral (Supplementary Figure S2 and Table S3) ensured the sutures were not fully ossified. All components of the calvarium fragments shown were measured using metrology associated with polarized Raman spectroscopy presented, in detail, in Supplementary Table S3. Data were analyzed with unpaired *t*-test with two-tailed distribution. * *p* < 0.05, ** *p* < 0.01.

The Erf-insufficient (*Erf^loxP/−^*) mice exhibit a mild embryonic reduction in the ossification of calvarial bones followed by a later postnatal multiple suture closure phenotype that varies in severity and age of onset among individuals [11]. To document the onset and progression of the disease, we examined skulls of *Erf^loxP/+^* and *Erf^loxP/−^* mice starting from P15 up to P65 using microCT imaging. To minimize background-biased results, all analyzed mice were siblings resulting from *Erf^loxP/loxP^* X *Erf ^+/−^* crossing with both parents from a mixed 129/SV × C57BL/6 background. A bone development defect, including hypomineralization of frontal bones and a significant delay in the formation of the posterior frontal (PF) suture, was the most common trait observed in P15 *Erf^loxP/−^* mice (Figure 1A–C and Supplementary Table S1. Signs of synostosis became apparent at this stage in half of the animals tested, affecting primarily the coronal sutures. As development continued, the synostosis phenotype became more severe and was evident in all P65 mice with the PF and coronal sutures most affected by closure. Although the PF suture in *Erf^loxP/+^* mice at this stage was still well-detected, it displayed closures and deformities in the majority of *Erf^loxP/−^* mice. Interrogation of single-cell RNA sequencing data from coronal [24] and frontal [25] sutures for the correlation of Erf expression with other genes, as previously described [22], indicated the mediation of additional pathways in the frontal suture, notably involving signaling and motility (Supplementary Figure S1). To evaluate possible qualitative differences in the ossification of cranial sutures, we utilized Raman spectroscopy on mouse calvaria at P65. Analysis of the suture mineralization of phenotypically normal *Erf^loxP/+^* and synostotic *Erf^loxP^*^/−^ animals indicated that both mineral and matrix components were comparable, as indicated by the polarized Raman spectra (Figure 1D,E and Supplementary Figure S2). Consistent with synostosis, however, the mineral-to-matrix ratio and the crystallinity were increased in the ossified sutures from the *Erf^loxP/−^* mice (Figure 1E), indicating that Erf insufficiency may have a quantitative rather than a qualitative effect on the ossification process.

**Figure 2:**
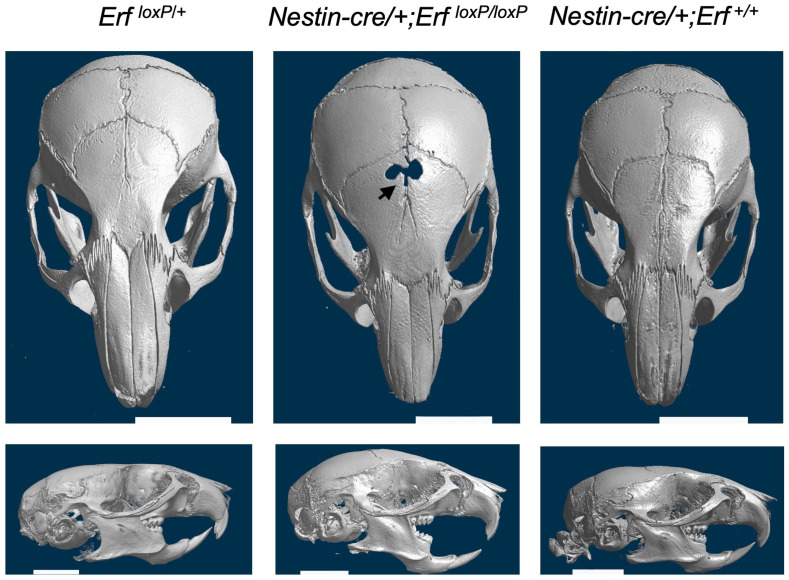
Elimination of Erf in cells of neuroectodermal origin causes a frontal bone development defect but not craniosynostosis. Representative skull microCT scans of *Nestin-cre/+;Erf^loxP/loxP^* P65 mice carrying homozygous deletion of Erf in cells of neuronal origin and age-matched *Nestin-cre/+* mouse. Although compromised development of frontal bones (black arrow) is evident in two of the three animals tested and facial deformity in all three animals, cranial sutures do not display synostosis. Cre recombinase activity in the neuronal tissues was verified on postnatal day 2 through crossing with the Gt(ROSA)26Sor^tm4(ACTB-tdTomato,-EGFP)Luo^ mouse line.

To gain insights into the physiology of the frontal bone development defect and the late-onset craniosynostosis, we initially examined the possible origin of the cells affected by Erf-insufficiency beyond the mesenchymal stem cells identified previously [22]. To evaluate the contribution of neural crest-derived cells that are contributing to suture formation [26], we generated *Nestin-cre/+;Erf^loxP/loxP^* transgenic mice in which Erf is specifically inactivated in neuronal progenitors and subsets of neuroectodermal mesenchymal stem cells. Interestingly, the microCT analysis of skulls at P65 showed an absence of craniosynostosis, yet the compromised development of frontal bones and a gap in the PF suture area was evident in two out of three animals obtained (Figure 2). Dental malocclusion and facial skeletal asymmetry were also observed in one of the three *Nestin-cre/+;Erf^loxP/loxP^* animals. Mice carrying the Nestin-cre transgene alone had a normal skull appearance indicating that the craniofacial phenotype of *Nestin-cre/+;Erf^loxP/loxP^* animals is attributed to apparent Erf elimination (Figure 2). These data suggest that Erf is required for the proper development of facial bones of neuroectodermal origin and that the initial ossification delay, as well as the variable craniosynostosis phenotype observed in mice, may be the result of Erf insufficiency in both mesodermal and neuroectodermal lineages and their interaction.

### 2.2. Effect of Mek1/2 and Xpo1 Inhibitors on Erf Localization and on Differentiating Primary Suture-Derived Cells

**Figure 3:**
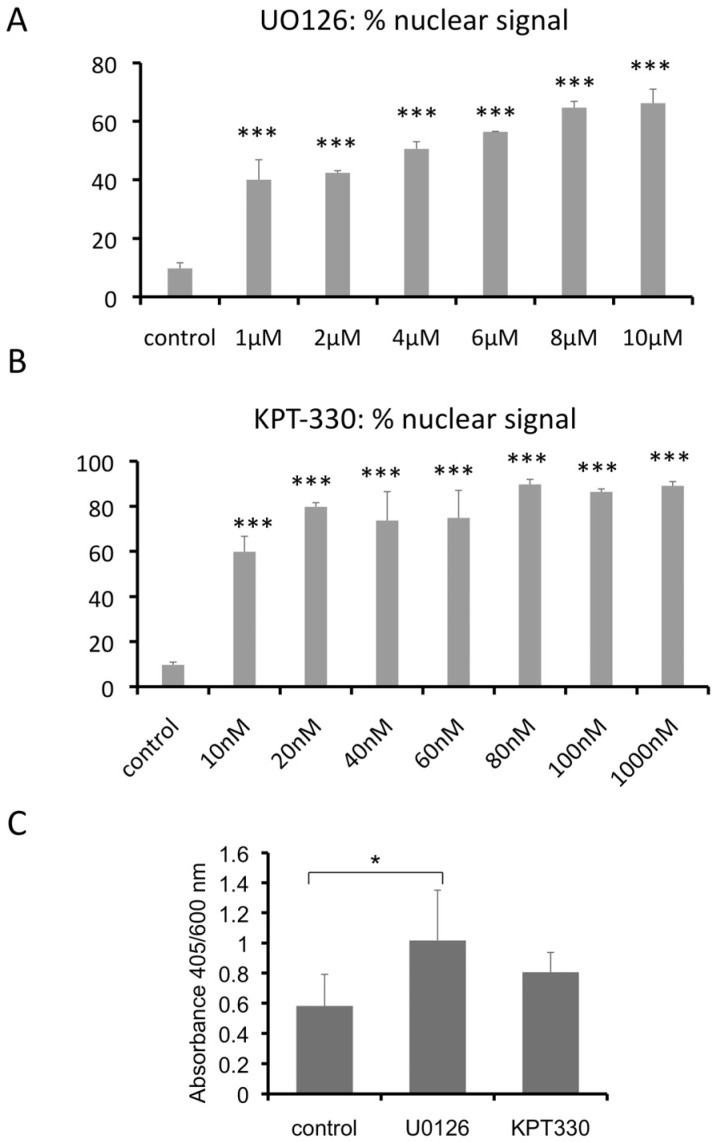
Nuclear accumulation of Erf after MEK1/2 or Xpo1 inhibition and the effect on osteogenic differentiation. Evaluation of the active concentration of inhibitors in cellular assays. (**A**,**B**) Mek1/2 inhibition by U0126 (**A**) and Xpo1 inhibition by KPT-330 (**B**) were evaluated by monitoring the subcellular localization of GFP-Erf in HeLa cells after 2 h exposure to the drugs. The average of at least 3 independent experiments is shown. (**C**) Calcium deposition per cell of cell cultures derived from coronal and sagittal sutures growing for 28 days in osteogenic medium in the presence of 2 μΜ U0126 and 10 nM KPT-330. Cell numbers were estimated using MTT assay by the absorbance at 600 nm and calcium deposits by the absorbance of Alizarin Red S at 405 nm after its extraction from the cells. Three independent biological experiments, with two experimental replicates each, were performed. In all cases, data were analyzed with one-way ANOVA followed by Dunnett’s (two-sided) test to compare all groups (treatments) against control group. * *p* < 0.05, *** *p* <0.001.

The apparent threshold effect of Erf in calvarial bone development and the presence of one functional allele in patients suggests that it could be a valid pharmacological target. We thus wished to explore the potential of Erf activity level modulation in our mouse craniosynostosis model to ameliorate the phenotype. In the absence of any cell-type-specific effect of Erf insufficiency that might have allowed a more targeted intervention, as well as the absence of any Erf-specific modulating compounds, we resorted to two wide-range inhibitors, which can affect Erf nuclear accumulation through independent mechanisms. We have shown that MEK1/2 inhibitors block Erf phosphorylation and permit its nuclear accumulation and transcriptional repressor activity [14]. Exportin inhibitors block Erf nuclear export, increasing its nuclear accumulation and transcriptional repression function, too [12,14,27]. We thus tested U0126, a specific MEK1/2 inhibitor that was also previously reported in the treatment of craniosynostosis in an Apert syndrome mouse model [7] and KPT-330, a nonreversible selective inhibitor of the nuclear exportin XPO1/CRM1 that is being tested as a potential antitumor drug [28,29,30]. Although both inhibitors affect multiple cellular processes, any common phenotypic changes could be suggestive of the modulation of Erf activity in craniosynostosis. Initially, we utilized an ex vivo assay using HeLa cells and determined the minimal concentration of the compounds that could achieve a significant nuclear accumulation of Erf as 1 μM and 10 nM for U0126 and KPT-330, respectively (Figure 3A,B). We further tested the cytostatic action of the compounds and evaluated the impact of nontoxic concentrations of 2 μM U0126 and 10 nM KPT-330 on the osteogenic differentiation of freshly isolated coronal and sagittal suture-derived primary cells (Figure 3C and Supplementary Figure S3). Even at these low concentrations, U0126 resulted in significantly increased mineralization per cell, while the seeming KPT-330 increase was not statistically significant. These data suggest that the inhibitors may be able to affect the mineralization process without affecting cell growth.

### 2.3. Local Administration of U0126 and KPT-330 Compounds in Mice Does Not Have Serious System-Wide Effects

**Figure 4:**
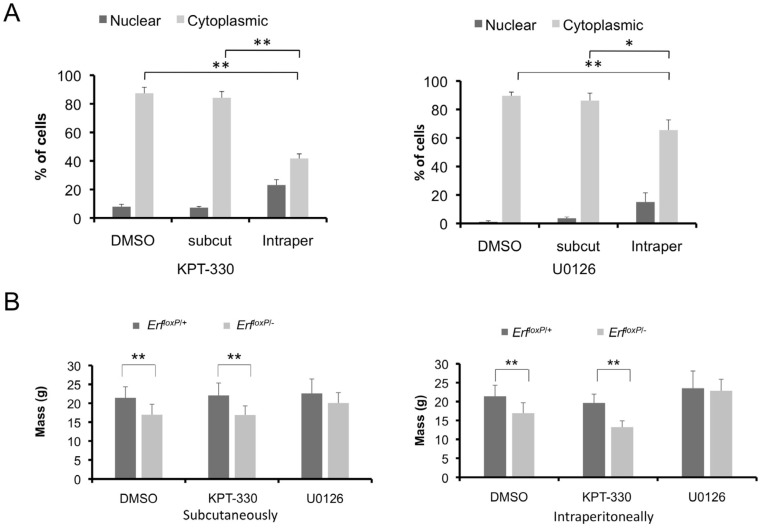
Local administration of Mek1/2 and Xpo1 inhibitors does not have system-wide effects. (**A**) Animals were injected either with 0.5 mg/kg of the inhibitors subcutaneously (subcut) over the skull or 50% DMSO or with 5 mg/kg of the inhibitors intraperitoneally (Intraper), per animal treatment protocol. Blood was collected after 6 h without sacrificing the animals, and the effect of the inhibitors was evaluated by the localization of GFP-Erf after the addition of 1% mouse serum in the cell cultures. KPT-330, (**left panel**). U0126, (**right panel**). The experiment was performed in triplicate. * *p* < 0.05, ** *p* < 0.01. (**B**) Effect on the weight of the animals at P65 per treatment protocol with 0.5 mg/kg of the inhibitors subcutaneously over the skull (**left panel**) or 5 mg/kg of the inhibitors intraperitoneally (**right panel**). Seven to ten animals were included in each group, and the data were analyzed using one-way ANOVA followed by Bonferroni correction for comparisons among the groups and unpaired (two-sided) *t*-test for comparisons between the two genotypes in each group; * *p* < 0.05, ** *p* < 0.01.

To examine whether a possible pharmacological augmentation of Erf localization in vivo could affect the craniosynostosis phenotype, we treated *Erf^loxP/+^* and *Erf^loxP/−^* littermates with U0126, KPT-330 or 50% DMSO carrier solution on alternate days from P5 to P65. To minimize possible toxic or secondary effects, we treated animals either systemically or locally with amounts consistent with previously used levels of the compounds: systemically via the intraperitoneal administration of 5 mg of either compound per kg of body weight or locally, with subcutaneous administration over the calvaria of 0.5 mg per kg. We utilized the HeLa cells ex vivo system above to evaluate the presence of the compounds in the bloodstream 6 and 24 h after injection. Indeed, no effective amounts of the drugs that could affect Erf localization were detected after local administration (Figure 4A). In contrast, intraperitoneal administration of the inhibitors could be readily detected in the bloodstream 6 h after injection but not 24 h after injection. An additional indication of drug presence and effect was the changes in the overall animal mass. Erf-insufficient animals are generally smaller than their siblings. Treatment with the MEK1/2 inhibitor U0126 intraperitoneally eliminated this difference (Figure 4B—right panel). In contrast, treatment with the exportin inhibitor KPT-330 did not affect the differences between the two genotypes; however, it seemed to cause an inhibition in the growth of Erf^loxP/−^ mice but without statistical significance (91.9% confidence). Local calvarium administration (Figure 4B—left panel) did not appear to affect the animal mass, though differences between the two genotypes were decreased in both U0126 treatment groups. Collectively, these data indicate that the treatments have a biological effect but also caution about possible secondary effects, especially in the case of systemic inhibitor administration.

### 2.4. Mek1/2 and Xpo1 Inhibitor Administration Alleviates Suture Closure and Restores Skull Morphology in Erf^loxP/−^ Mice

**Figure 5:**
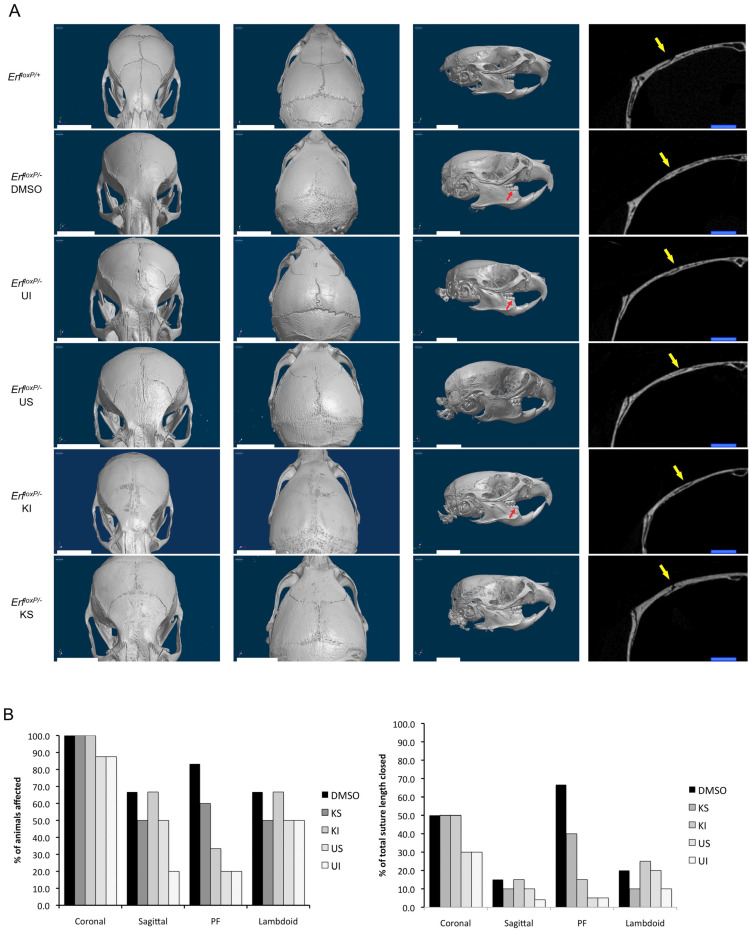
Mek1/2 and Xpo1 inhibitors can ameliorate Erf-related craniosynostosis. (**A**) Volume renderings (3 images on the left) and transverse section image midway through the right coronal suture (rightmost image) derived from microCT scans of representative Erf-competent (*Erf^loxP^*^/+^) and Erf-insufficient (*Erf^loxP/^*^−^) animals at P65. Littermates were treated intraperitoneally on alternate days from P5 to P65 with 5 mg/kg of U0126 (UI) or KPT-330 (KI) or subcutaneously with 0.5 mg/kg of U0126 (US) or KPT-330 (KS) over the skull or with the inhibitor solvent only (DMSO). All animals in a litter received the same treatment. Dorsal anterior (left), dorsal posterior (middle) and lateral (right) images of representative animal skulls are shown. Volumetric images were constructed using the same opacity and luminescence settings. Red arrows indicate animals with dental malocclusion. Yellow arrows indicate the coronal suture. White scale bars, 5 mm; blue scale bars, 1 mm. (**B**) Evaluation of suture synostosis from the microCT scans of *Erf^loxp/−^* treated animals. The percentage of mice exhibiting a defect in the indicated sutures for each treatment group is shown in the left panel. The right panel indicates the percentage of the total length of each suture that was fully ossified, considering all the animals in each group. The evaluation was performed independently by two scientists blind to genotype and treatment. Each measurement is based on four mice, with the exception of KI treatment group, which includes three mice and the DMSO group, which includes six mice. All groups have one female mouse except the DMSO group, which has two.

To evaluate the effects of the treatment with the MEK1/2 U0126 and Exportin KPT-330 inhibitors on Erf-related craniosynostosis, microCT scans of the skulls were examined and visually evaluated at the end of the treatment period (Supplementary Figure S4). Both inhibitors and treatments appeared to ameliorate synostosis, as evaluated independently by two observers. Both the percentage of mice displaying affected sutures and the extent of suture closure were scored (Figure 5). *Erf^loxP/−^* mice treated either locally or systemically with KPT-330 or U0126 exhibited a clear improvement in the extent of synostosis, though none of the treated animals was identical to their *Erf^loxP/+^* siblings. DMSO-treated Erf-insufficient mice had extensive synostosis in the coronal, lambdoid, sagittal, posterior frontal (PF), accessory occipital and even the frontonasal sutures, consistent with the multiple suture synostosis phenotype described previously [11]. In addition, the interfrontal bone was undetectable, and the mice exhibited pronounced dental malocclusion. The inhibitor-treated animals showed a variable degree of improvement. The coronal sutures were still affected in most treated animals, yet the extent of ossification was decreased. Particularly the PF and sagittal sutures displayed the greatest improvement across the mice analyzed in the study. Intraperitoneal treatment with the MEK inhibitor U0126 appeared to be the most effective, while intraperitoneal treatment with the Xpo1 inhibitor KPT-330 was the least effective (Figure 5B). The treated *Erf^loxp/+^* control animals exhibited no defects beyond an accelerated ossification in the PF suture region and a morphological abnormality in the coronal suture region present in two out of the eight KPT-330-treated animals (Supplementary Figure S5). Interestingly, subcutaneous administration of the inhibitors but neither intraperitoneal administration of the inhibitors nor subcutaneous administration of the DMSO solvent solution resulted in a significant increase in skull thickness (Supplementary Figure S6). The apparently enhanced bone formation in the presence of inhibitors is consistent with the role of Erf on bone development and the mineralization defect of frontal bones observed upon Erf insufficiency.

**Figure 6:**
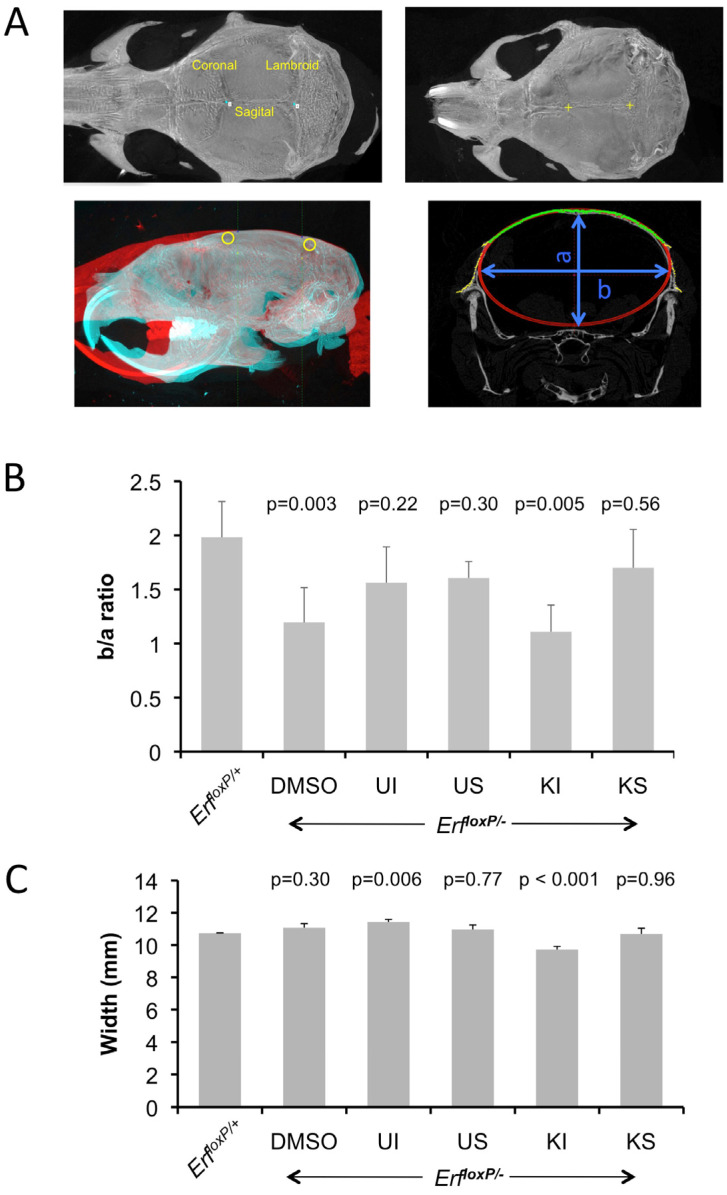
Mek1/2 U0126 and Xpo1 KPT-330 inhibitors improve skull morphology of the *Erf^loxP/−^* mice. (**A**) For the semiautomated analysis of the curvature of the skull, the lambda and bregma points were marked on the volume renderings of the microCT scans (upper panels), and then all skulls were programmatically aligned to a reference normal skull (lower left image), and a transverse section at ¼ of the lambda/bregma axis (measured from the bregma) was used to fit the ellipse. Cranial sutures in top left panel are named in yellow letters. (**B**) Curvature of the skull as evaluated by the b/a ratio of the fitted ellipse. Average of 3–5 male animals per group treated as in (**A**) above. Data were analyzed with one-way ANOVA followed by Dunnett’s (two-sided) test to compare all groups (treatments) against control group (*Erf^loxP/^*^+^). (**C**) The overall width of the calvarium was measured directly from the microCT volume rendering with Bruker DataViewer 1.5.6.2 software.

To obtain a more quantitative and objective measurement of the changes, we developed and employed a semiautomated method to estimate the curvature of the cranium as an indicator of synostosis and skull morphology. We used bregma and lambda as reference points in each skull and analyzed microCT images corresponding to transverse sections taken at ¼ of the bregma–lambda axis from the bregma (Supplementary Figure S7). By automatically fitting an ellipse to each image, we calculated the b/a ratio, in which a and b represent the minor and major axis lengths of the ellipse quadrant, respectively (Figure 6). Consistent with our visual observation, the decreased b/a ratio corresponding to decreased ellipticity of the Erf^loxP/−^ calvaria is eliminated after treatment with the compounds without affecting the overall width of the cranium (Figure 6B,C), with the exception of the intraperitoneal treatment with KPT-330. Collectively, these data indicate that the modulation of Erf nuclear levels by both inhibitors that can augment Erf function may contribute to the amelioration of the craniosynostosis phenotype in Erf^loxP/−^ mice.

## 3. Discussion

Syndromic craniosynostosis due to *ERF* haploinsufficiency presents some unique challenges and opportunities for disease understanding and management. Clinically, the phenotype shows overlap with FGFR2-driven craniosynostosis, usually Crouzon syndrome [10,31]. This is not surprising as FGFR activation leads, among other effects, to ERF phosphorylation, nuclear export and functional inactivation. In contrast to FGFR-driven craniosynostosis syndromes, however, CRS4 has an unusual late-onset phenotype with variable severity, indicating that small quantitative and/or genetic background differences may affect the Erf-deficiency phenotype. This is clearly evident in mice in which the 50% decrease in Erf levels of *Erf^+/−^* animals has no apparent effect on phenotype, but the 70% decrease observed in *Erf^loxP/−^* animals results in craniosynostosis. It would thus appear that Erf nuclear levels and transcriptional activity may be contributing factors not only in CRS4 but also in other forms of RTK receptor-driven craniosynostosis.

In contrast to humans, in whom the sagittal and lambdoid sutures are predominantly fused [10,32], *Erf^loxP/−^* mice present defects primarily in coronal sutures (100% prevalence), but also all other cranial sutures are affected by the end of the growth. Interestingly, at postnatal day 15 (P15) before craniosynostosis has fully developed, reduced growth of the frontal bones resulting in the delayed formation of the PF suture is the most common characteristic of Erf-insufficient mice. In mouse models of infantile hypophosphatasia and Crouzon syndrome, diminished cranial bone volume and hypoplasia have also been reported along with craniosynostosis [33,34,35]. Although premature suture fusions and mechanical signals can cause compensatory overgrowth or alterations to neighboring sutures and bones, the frontal bone development defect observed in *Nestin-cre/+;Erf^LoxP/LoxP^* mice that do not display craniosynostosis indicates that Erf may have a direct effect on the formation of neural crest-derived frontal and facial bones. Indeed analysis of scRNA sequencing data indicates that Erf may participate in cell motility and growth regulation consistent with a previously reported role in cell motility [36]. The incomplete penetrance of the frontal bone defect is likely due to the inefficient homozygous deletion of both *Erf* alleles (unpublished observations). Thus, the overall craniosynostosis phenotype in *Erf^loxP/−^* mice seems to be the result of Erf insufficiency in both neuroectodermal and mesodermal lineages that may involve additional pathways in neuroectodermal origin cells. Further studies including conditional *Wnt1-cre* and *Mesp1-cre* mice [37] are needed in order to elucidate the potential dual role of Erf in craniofacial development.

Total *Erf* loss is detrimental [19,20], and the known human *ERF*-related pathologies manifest in the heterozygous state [11,16,17], allowing a fully functional allele for possible intervention. The mechanism of Erf regulation via phosphorylation-induced nucleocytoplasmic shuttling, as well as the absence of any gain-of-function ERF mutations in craniosynostosis and other Erf-related pathologies, makes Erf a worthy pharmacological target. In the absence of Erf-specific compounds, we tested this hypothesis with alternative compounds. U0126, a specific MEK1/2 inhibitor but low potency drug [38,39], was used with encouraging results in the treatment of an FGFR2-induced craniosynostosis mouse model [7], and we have shown that it can effectively regulate Erf localization and function [14]. Nuclear export-targeting Xpo1 inhibitors have also been shown to regulate Erf localization and function [12]. KPT-330 is a potent nonreversible Xpo1 inhibitor currently in clinical trials in cancer therapy [28,29,40].

Treatment with either inhibitor ameliorated the synostosis phenotype in Erf-insufficient animals. However, no animal appeared completely normal at the end of the treatment period. It is possible that the decreasing in vivo levels of the drugs during the 48 h period between treatments may not maintain sufficient nuclear Erf levels during the entire treatment period. Indeed, the drugs were not detectable in the bloodstream 24 h after injection in our assays, though their effect on Erf cannot be excluded. It is also possible that the opposite, the complete inhibition of the physiological Erf shuttling and the resulting transcriptional derepression at certain time points, may be required for proper cell fate regulation [41]. We have previously shown that nuclear Erf mutants have detrimental effects on fibroblast and epithelial cell proliferation [15,42]. Thus, growth delays could have affected the amelioration of the phenotype, delaying the expansion of the required cell types and countering possible effects on improved differentiation potential. Finally, the pleiotropic effects of both the MEK1/2 and Exportin inhibition should be considered. Erk is known to have both positive and negative roles in osteogenic differentiation, and constant inhibition of its function can obscure the therapeutic effects [43,44,45,46,47], particularly as Erf levels and activity do not affect Erk activity [11,42]. The role of nuclear export in osteogenic differentiation cannot be excluded either, as there is some indication that it may affect osteoclast differentiation [48]. Exportin inhibition may also lead to the nuclear accumulation of a wide range of cellular factors affecting the process in multiple ways. However, the fact that both inhibitors (U0126 and KPT-330) that independently enhance Erf nuclear presence ameliorate the *Erf^loxP/−^* craniofacial phenotype indicates that the improvement can be attributed, at least in part, to the augmentation of the remaining Erf transcriptional repressor function.

It is particularly interesting and unique to our knowledge that local subcutaneous administration of the drugs at an order of magnitude lower dosage had a comparable effect on synostosis. This suggests that the effects of the compounds on suture development are direct, affecting proximal cells, and are less likely to involve signals from other systems triggered by Erf activity or pathway inhibition. The increased in vitro cell mineralization and skull thickness after U0126 and KPT-330 treatments are consistent with the previously reported positive role of Erf in mesenchymal suture cell osteogenic differentiation [22] and the decreased frontal bone formation we observed in the mouse models upon Erf insufficiency. It is also encouraging that no system-wide effects were apparent after local administration. The XPO1 inhibitor, in particular, is a potent cytostatic antitumor compound that was likely to have some effect even at the very low concentrations used here. The local administration minimized this possibility and allowed the evaluation of its effect on synostosis.

The use of strong antitumor compounds, even when locally applied, would be challenging to justify in pediatric syndromes. However, our data highlight the potential of Erf as an intervention target, as well as the feasibility of local treatments. At the same time, they point to the limitations of the currently available modulators of ERF function. The precise control of Erf levels seems to be crucial for cranial suture formation and bone development originating from both neuroectodermal and mesodermal lineages. The role of Erf in each cell lineage and in individual cell types and at different developmental time points remains to be further explored. Identification or development of more specific activators of the residual ERF suppressor function could be a potent approach not only in ERF-related craniosynostosis but likely in other FGF/MAPK-related craniosynostosis disorders. Such a treatment, either at early stages or postoperatively, could prevent multiple surgical interventions and associated complications [3,4,49], increasing the welfare of the patients.

## 4. Materials and Methods

### 4.1. Mouse Lines

Mice were bred and maintained in the animal facility of the Institute of Molecular Biology and Biotechnology in Greece. All experimental protocols were conducted within ethical guidelines and in compliance with the 3Rs. Protocols were approved by the bioethics committee of IMBB and licensed from the General Directorate of Veterinary Services, Region Crete (permit numbers EL 91BIO-02 and EL91-BIOexp-02; project license Nos. 27289/2014, 93137/2018 and 106346/2021 to G. Mavrothalassitis). *Erf^loxP/+^* and *Erf^loxP/−^* littermates were obtained by crossing *Erf^+/−^* mice with *Erf^loxP/loxP^* mice, both of which have been reported in the literature [11]. The B6.Cg-Tg(Nes-cre)1Kln/J mice [50] were from The Jackson Laboratory. Effective activation of cre recombinase was detected in neuronal origin cells as early as P2. All animals were of a mixed 129/SV × C57BL/6 background.

### 4.2. Cell Lines and Transfection

HeLa cells were cultured in DMEM low glucose medium (Gibco by Thermo Scientific, Waltham, MA, USA, 21885025) supplemented with 10% FBS and 100 U/mL penicillin/streptomycin. The cells were transfected with the GFP-ERF fusion plasmid DNA using the calcium phosphate protocol, as described previously [14]. At the end of each experiment, cells were fixed with cold methanol–acetone (1:1) solution for 10 min at −20 °C, and ERF subcellular localization was studied through the detection of GFP protein autofluorescence.

### 4.3. Suture Cell Culture and Differentiation Assay

Freshly isolated cranial suture cells were obtained from 5-day-old mice, as described previously [22,51,52]. Osteogenesis was induced in early passage cultures at approximately 70% confluency by the addition of DMEM low glucose medium (Gibco by Thermo Scientific 21885025) supplemented with 10% FBS, 0.1 μM dexamethasone, 50 μM ascorbate-2-phosphate and 10 mM β-glycerophosphate. In the experiments with inhibitors, the freshly prepared osteogenic differentiation medium was supplemented with either 2 μM U0126 (Cell Guidance Systems SM106, St. Louis, MO, USA) or 10 nM KPT-330 (synthesized as described below) before every medium change. The extent of osteogenic differentiation was evaluated using Alizarin Red S (Sigma A-5533 Sigma-Aldrich, Saint Louis, MO, USA) staining of the cultures and measured using acetic acid extraction of the dye and quantification at 405 nm, as already described [53].

### 4.4. KPT-330 Preparation

KPT-330 was prepared according to a literature procedure [54] with minor modifications, as shown in Supplementary Figure S8.

### 4.5. Administration of Inhibitors in Animals and Skeletal Preparation

U0126 and KPT-330 inhibitors were administered in newborn mice either by skull subcutaneous or intraperitoneal injections on alternate days starting from postnatal day 5 (P5) up to P60. For the skull subcutaneous treatment, 0.5 mg of U0126 per kg of body weight diluted in DMSO-PBS (1:1) solution and 0.5 mg of KPT-330 per kg of body weight diluted in DMSO alone were used. The volume of the injected solution did not exceed 4 μL. For the intraperitoneal treatment, 5 mg of either U0126 or KPT-330 per kg of body weight was diluted into DMSO-PBS (1:1) solution and injected into mice. The control groups were injected with DMSO-PBS (1:1) solution. At the end of each treatment, animals were sacrificed, and skulls were placed in 70% ethanol overnight with gentle agitation, followed by change to 95% ethanol for further fixation and storage.

### 4.6. MicroCT Analysis

MicroCT scans were performed at the Hellenic Centre for Marine Research (HCMR) using a Skyscan 1172 microtomograph (Bruker, Kontich, Belgium). This scanner uses a tungsten X-ray source equipped with an 11PM CCD camera (4000 × 2672 pixels). All specimens were scanned at a voltage of 75 kV and 131 μA with an aluminum filter of 0.5 mm and a pixel size of 13.79 μm for a half rotation of 180°. Projection images were reconstructed into cross-sectional images using SkyScan NRecon 1.7.4.2 software (NRecon, Bruker, Kontich, Belgium), which implements a modified Feldkamp back-projection algorithm. All scans were rotated upright in the software DataViewer (DataViewer 1.5.6.2, Bruker, Kontich, Belgium), and subsequently, the new datasets were loaded into the software CT Analyser v.1.18.4.0. The calvaria thickness was calculated for each sample through the manual creation of a volume of interest (VOI) between the lambdoid and frontonasal sutures and a custom processing plugin of CTAn 1.18.8.0 software (Supplementary Figure S6).

Suture fusion was evaluated by two researchers from genotype blind microCT dorsal, posterior dorsal, anterior dorsal and inferior images, as shown in Supplementary Figure S4. For consistency between the evaluators, the left and right coronal sutures were considered in 3 parts, the left and right lambdoid and the sagittal in 5 parts and the posterior frontal in 2 parts. The sagittal, for example, could be fused for the 0–5 fifths of its length, each coronal for 0–3 thirds and so on. There were no discrepancies between the two evaluators.

### 4.7. Calvaria Morphometric Analysis

We built a global descriptor that would characterize the cranium curvature based on the fitting of an ellipsis on the coronal slices extracted from the microCT of the skulls. To do so, we initially needed to remove the bias from the overall size variations and positioning inconsistencies among the different scans. Two landmarks were placed in the lambda and bregma points on each skull (Supplementary Figure S7), and a semiautomatic process was used to calculate shape-preserving transformations that would align the chosen landmarks in all the skulls. The fitting process was devised to minimize size variations and positioning inconsistencies among different skulls. All the measured skulls in a preprocessing step were converted from the 16-bit output of the scanner to 8-bit to reduce memory requirements and processing time. Using rotations on the volumes, the skulls were coaligned along the three main axes—*x*-axis (dorsoventral), *y*-axis (mediolateral) and *z*-axis (medial). One of the skulls was randomly assigned as the reference skull, and in the next steps, the bregma–lambda lines of all the skulls were superimposed to this reference skull. With the input from an independent expert, the bregma and lambda landmarks were selected on the horizontal plane view (*y*–*z* plane) for reference and all the other skulls, and the landmarks were stored for further processing.

Using the stored bregma and lambda landmarks, an optimal transformation was calculated and applied in the horizontal plane for each skull so that an alignment of the respective landmarks for each skull to the reference was achieved. This transformation was constrained to translations, rotations and uniform scaling of the skulls so that the overall shapes in the moving skulls remained unchanged. The transformations were calculated in the two-dimensional horizontal plane but applied to the whole three-dimensional volumetric images. The height (*x*-axis) of the lambda and bregma landmarks was recovered with an automatic process using edge detection on the grayscale values of the volumetric images, and a secondary 2D transformation was calculated on the sagittal plane (*x*–*z* plane). This completed the alignment of the respective three-dimensional lambda and bregma landmarks between each skull and the reference skull. Using the superimposed bregma–lambda line as a reference, a slice of the skull along the coronal plane (*x*–*y* plane) was extracted perpendicular to the bregma–lambda line and at a distance equivalent to that of ¼ starting from the bregma landmark.

Using the extracted slices for each skull, the lateral edges of the parietal bones were marked, and a transformation, constrained only to translations, rotations and uniform scaling of the skulls, was calculated so that the individual skull landmarks aligned with those of the reference skull. A cloud of points was collected along the outside of the surface for each skull ranging from the left to the right lateral edges of the parietal bones using edge detection in the grayscale values along the *x*-axis (green in Supplementary Figure S7). The points cloud was used in a least squares estimator that would fit an ellipse (red in Supplementary Figure S7), such as Ellipse:: ((X − X_0_)/a)^2^ + ((Y − Y_0_)/b)^2^ = 1, where (X_0_,Y_0_) is the center of the ellipse, and a, b are the ellipse “radiuses” (or subaxes) (blue in Supplementary Figure S7). The two subaxes of the fitted ellipse, determined by calculating the b/a ratio, can quantify the roundness of the ellipse as the measure of how closely the shape of the ellipse approaches that of a perfect circle (e.g., a/b = 1).

To verify the selection of the specific slice upon which we fit the ellipsis, we tried out several different slices perpendicular to the bregma–lambda line at multiple distances from the bregma point. A perpendicular slice at ¼ of the distance starting from the bregma produced the most robust and clear results in each case and, therefore, was chosen for the descriptor. In addition, different declinations from the perpendicular plane to up to 5 degrees in each direction between the lambda–bregma line and the plane of the examined slice were collected and examined by calculating the descriptor. It was found that the introduced error naturally had a small effect on the actual value of the skull’s indicator of synostosis, but the characterization outcome remained robust to the introduced error.

Moreover, in this semiautomatic process, whenever human input was required, the process was repeated at least five times to certify that the expert’s choice of landmarks had minimal and insignificant bias on the final classification of the skulls. The relevant MATLAB code and examples can be downloaded from https://cloud.iesl.forth.gr/index.php/s/MrLbztJWGWoiwmg, accessed on 8 March 2023.

### 4.8. Raman Spectroscopic Analysis

Polarized Raman measurements were carried out on a Renishaw inVia Reflex Raman microscope with a 785 nm excitation laser line with output power of 190 mW. The system was equipped with a half wavelength in the laser beam path and a polarizer in the path of the collected Raman signal (Supplementary Figure S2). A Renishaw translation XYZ stage with spatial resolution of 0.1 μm allowed the rapid line mapping by collecting Raman spectra in the spectral region 760–1020 cm^−1^. A large working distance microscope objective Leica x20 was used to focus the laser beam onto the specimen (after drying from ethanol), producing a laser power of ~37 mW to ensure the detection of low-intensity vibrational modes of the bone tissue.

Three line maps separated by 500 μm were conducted in three random locations on the bone (Supplementary Figure S2B). Each line map consisted of ~30 polarized Raman spectra spaced 10 μm apart. The total acquisition time for each line map was approximately 6 h, and their elaboration was accomplished using Renishaw Wire 4.2 software.

Regarding the configuration of laser beam and collected Raman signal polarizations, the importance of polarization direction with respect to the suture was studied. In particular, we identified that characteristic Raman band ratios were more sensitive to the collagen fibril orientation of the suture in the transversal direction relative to it. To this end, we chose the transversal configuration zz, which corresponds to polarization configurations of both laser and collected Raman signals perpendicular to the *x*-axis, the orientation of the suture (Supplementary Figure S2A,B).

For each Raman spectrum acquired, eight distinct Raman peaks were fitted with a mixed Gaussian/Lorentzian profile in the range from 830 to 1020 cm^−1^ (Supplementary Figure S9). Within this region, the bands in the polarized Raman spectrum associated with minerals and matrix of bone tissue and their assignments [55,56] are summarized in Supplementary Table S2. From the fitted peak profile characteristics (peak position, height, full width at half maximum and area under the fitted curve), the ratios summarized in Supplementary Table S3 were calculated.

In order to ensure a proper characterization of the suture region, the experimental protocol consisted of three important steps: (1) Polarized Raman maps were acquired along a line perpendicular to the suture at three random regions on the bone covering the whole width of the suture (Supplementary Figure S2B). (2) The fitting of the spectra was performed, and the Raman spectra that yielded the lowest value(s) of the ratio I_961_/I_1005_ were the ones used to safely represent the suture region, showing that the collagen content was most prominent in both healthy and craniosynostotic samples [57]. (3) These specific Raman spectra were elaborated for the calculation of the mean values of the parameters summarized in Supplementary Table S2.

### 4.9. Single-Cell Correlation Analysis

Single-cell correlation analysis was performed, as previously described [22], utilizing published single-cell RNA sequencing data from coronal (GEO accession number GSE163693) and frontal (Facebase BIOSAMPLE: 1-8DVM (scWFE18_S631) and 1-P586 (scWFE16_S598)) mouse sutures [24,25]. Briefly, count matrices sequencing data were filtered for quality, normalized and cleared of genes not detected in at least 2% of the cells. Positive and negative gene correlations (Supplementary Tables S4 and S5, respectively) with a false discovery rate at 0.05 significance were identified and further evaluated with the Wilcoxon rank sum test for their distribution in cells expressing the target gene or not. Enrichment analysis sets for *Mus musculus* were performed with gprofiler2. Clustering of correlated gene sets across different scRNA datasets was visualized with the Metascape web tool [58].

### 4.10. Statistical Analysis

Unless otherwise stated, the experimental data were analyzed using SPSS v19 software. Unpaired (two-sided) *t*-test was performed for comparisons between two groups, and one-way analysis of variance (ANOVA) was used for multiple comparisons, followed by post hoc Dunnett’s two-sided test or Bonferroni correction according to the experimental requirements. Levels of significance: * *p* < 0.05, ** *p* < 0.01, *** *p* <0.001.

## 5. Conclusions

Our analysis indicates that *Erf* insufficiency affects both neuroectodermal and mesodermal cells leading to late-onset craniosynostosis (OMIM 600775; craniosynostosis 4 (CRS4)). The phenotype can be ameliorated by the topical administration of compounds that enhance the residual Erf activity, suggesting a promising approach for intervention in CRS4 and possibly other RTK pathway-triggered syndromic craniosynostosis.

## Data Availability

The data presented in this study are available in Supplementary Materials, as well as publicly accessible links and references provided within the manuscript.

## Supplementary Materials

The following supporting information can be downloaded at: https://www.mdpi.com/article/10.3390/ijms24097961/s1.
