# Supplementary material for: Development of Erf-Mediated Craniosynostosis and Pharmacological Amelioration
Source: Int J Mol Sci. 2023 Apr 27;24(9):7961. doi: 10.3390/ijms24097961 (PMC10178537; doi:10.3390/ijms24097961)
Supplement: Supplementary file 1 [file ijms-24-07961-s001.zip › ijms-2303085-supplementary.pdf]

Supplementary figures, figure legends and supplementary tables 2 & 3

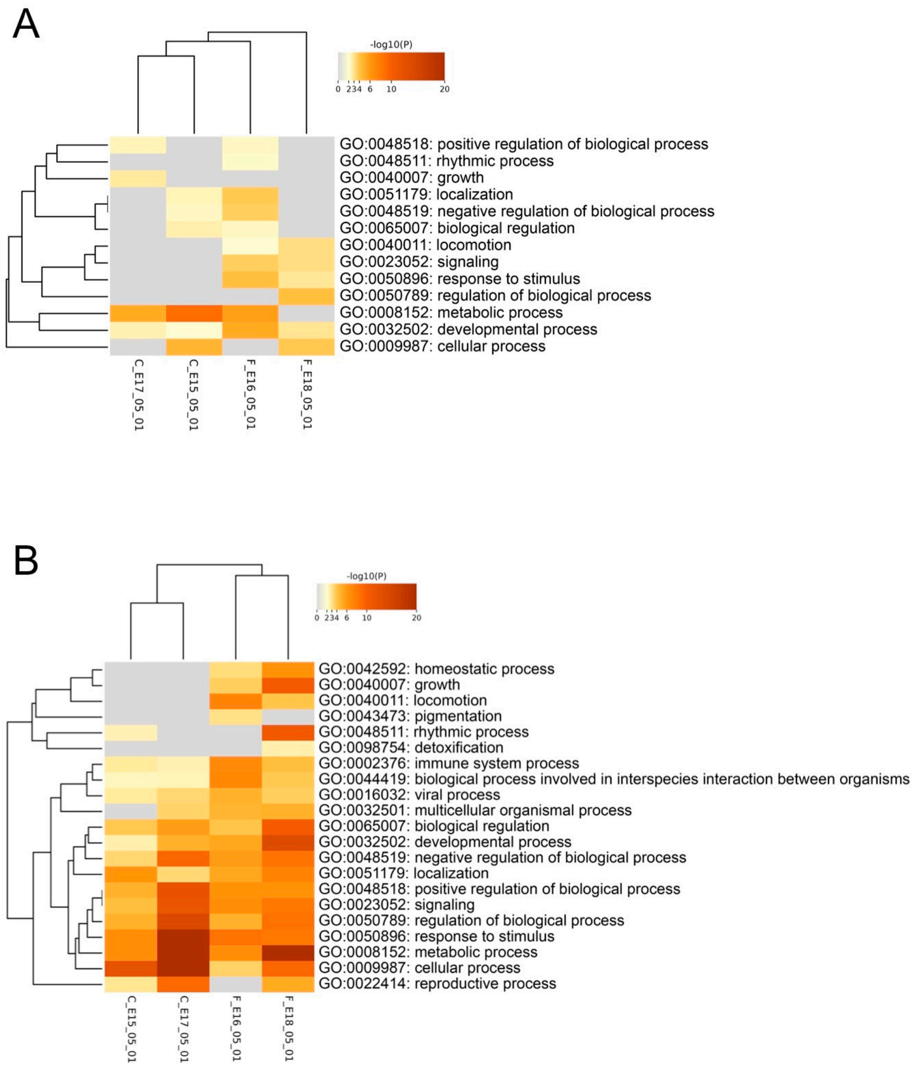

Supplementary Figure S1: Erf expression correlates with distinct gene sets in metopic and coronal suture. Cellular processes of genes whose expression positively (A) and negatively (B) correlates with *Erf* expression in single cell mRNA analysis experiments (see Supplementary Tables 4 and 5) from coronal (C_) and frontal (F_) sutures at embryonic day 15-18 (E<number>_). Segregation follows suture origin and genes from the frontal suture associate primarily with motility and growth.

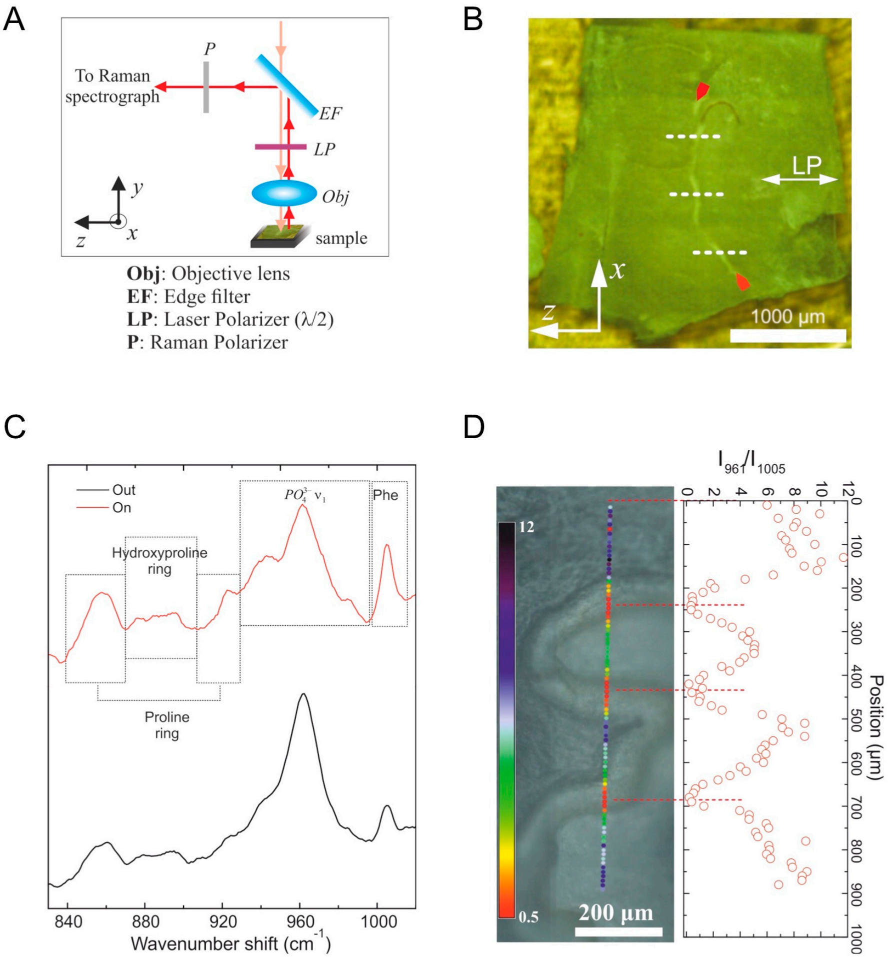

Supplementary Figure S2: Raman spectrum acquisition method for the analysis of calvarial bone and suture mineralization. A) Schematic representation of the experimental set-up showing the sample orientation and the polarization of both excitation and collected Raman signal. B) Indicative optical microscopy image of a specimen. The two edges of the suture are marked by the red arrows and the polarization of the excitation radiation (*z*) relative to the suture orientation(*x*) is also shown. The three dashed lines (not to scale) are the Raman mapping lines separated by 500 μm. C) Indicative polarized Raman spectra in the region 780-1020 cm^{-1} recorded onto (red line) and out of the suture (black line). Polarized Raman spectra were acquired in the transversal configuration zz corresponding to polarization configurations of both laser and collected Raman signal perpendicular to *x*-axis. The boxes indicate Raman bands assigned to proline ring, hydroxyproline ring and phenylalanine (Phe) and related to collagen. The phosphate band (ν_1 PO_4^{3-}) indicates the mineralized tissue. See also Supplementary Tables 2 and 3. D)

Indicative heat line map (to scale) of the ratio of intensities at 961 and 1005 cm^{-1} I_{961}/I_{1005} crossing a suture (left panel) and the corresponding graph of the ratio vs position in μm (right panel).

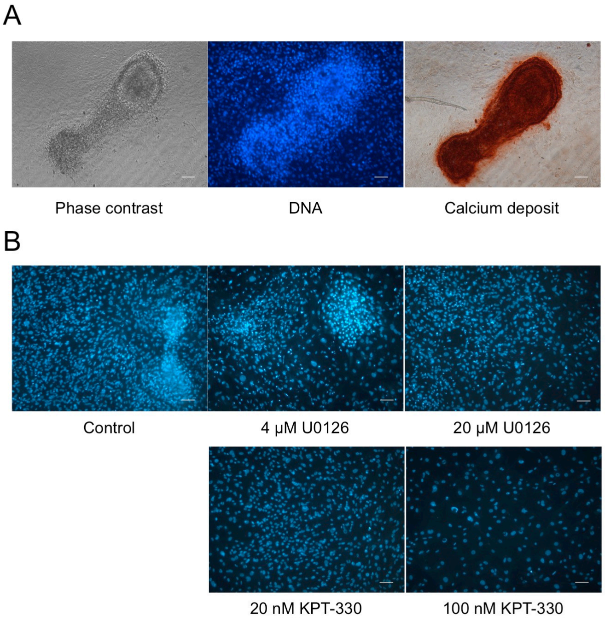

Supplementary Figure 3

Supplementary Figure S3: Mek1/2 and Xpo1 inhibitors can affect proliferation of differentiating cranial suture cells. A) Microphotographs of a nodule of suture derived cells growing for 21 days in osteogenic medium. DNA was stained with Hoechst-33342 and calcium deposit with Alizarin Red S. Scale bars: 100μm. B) Microphotographs of cells growing for 28 days in osteogenic medium in the presence of the indicated concentrations of the Mek1/2 and Xpo1 inhibitors showing growth inhibition at increased concentration. Scale bars: 100μm.

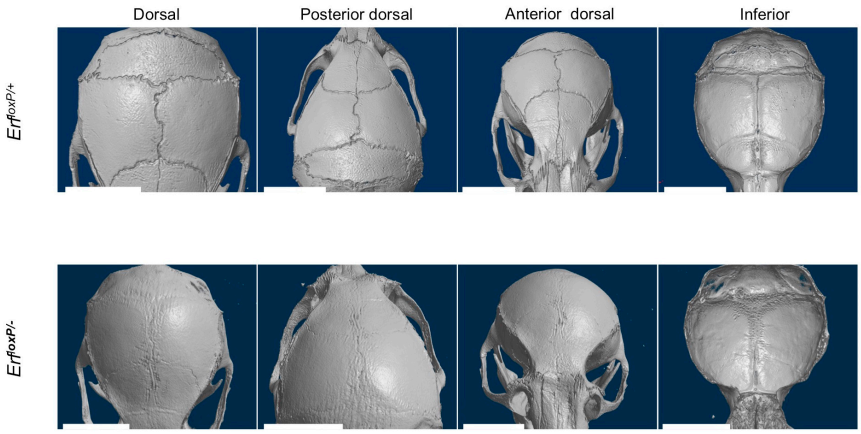

Supplementary Figure S4: Suture synostosis evaluation image set. Representative sets of four images for each calvarium derived from microCT

scans that were used to visually evaluate the extent of synostosis in P65 animals. The images were generated and captured with the Bruker CTvox volume rendering software. White scale bars, 5mm.

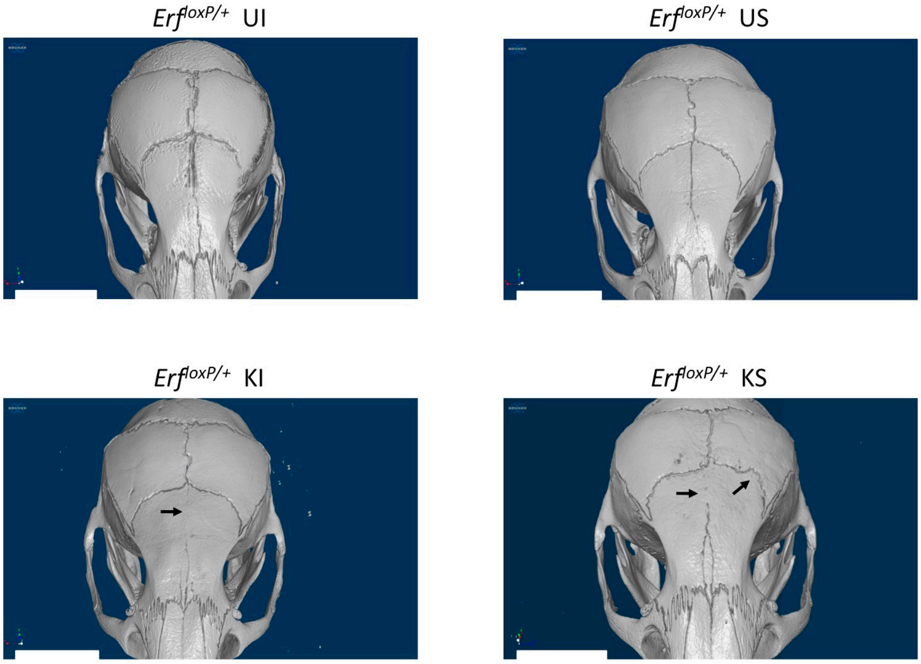

Supplementary Figure S5: Mek and Xpo1 inhibitors can rarely affect suture ossification in *Erf*$^{loxP/+}$ control mice. Volume renderings derived from microCT scans of the control *Erf*$^{loxP/+}$ P65 mice, treated with U0126 (U) or KPT-330 (K) intraperitoneally (I) or subcutaneously (S). In 2 out of 8 kpt330-treated animals, the posterior frontal and/or the coronal sutures appear affected (indicated by arrows). White scale bars, 5mm

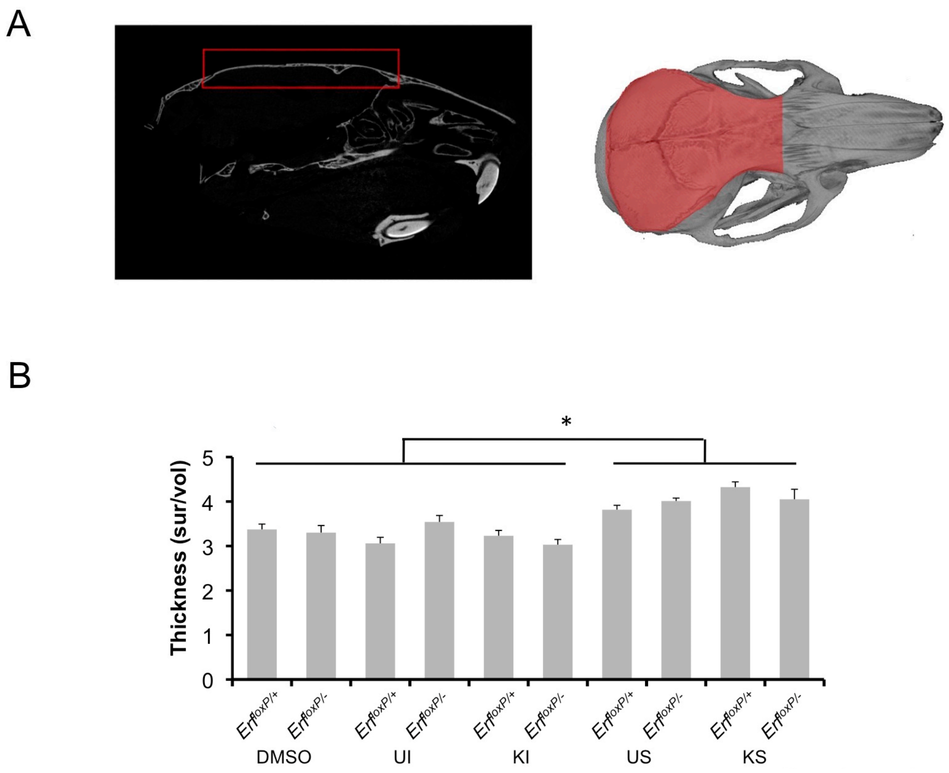

Supplementary Figure S6: Subcutaneous administration of Mek and Xpo1 inhibitors increases skull thickness. A) The area of the skull between the lambdoid and the frontonasal sutures (left panel) was isolated to form a volume of interest (VOI) shown in red (right panel) used to calculate calvarium thickness. B) Treatment of the animals with subcutaneous injection of the inhibitors U0126 (US) and KPT-330 (KS) but not DMSO or intraperitoneal administration of the inhibitors (UI and KI) increases skull thickness, independently of Erf level * $p < 0.05$

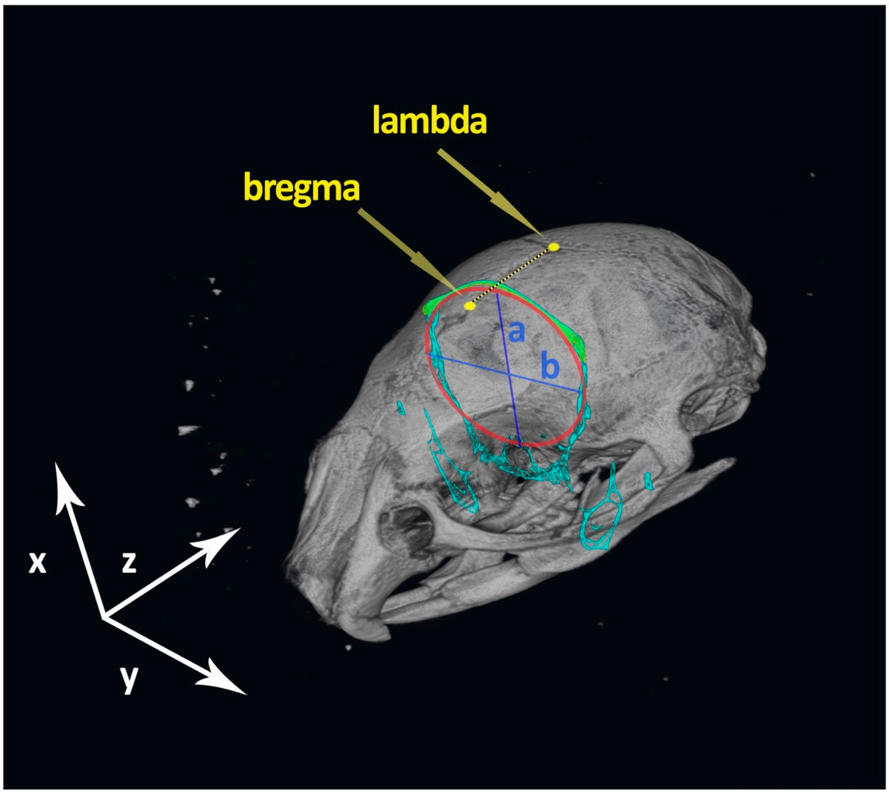

Supplementary Figure S7: Semi-automatic analysis of calvarium curvature by ellipse fitting. The bregma and lambda points of the skull, yellow arrows in the figure, are located in a semi-automatic manner that is used to define the bregma-lambda line, indicated by the yellow-black line in the figure. A coronal plane (x-y plane) is extracted perpendicular to the bregma-lambda line and at a distance equivalent to that of ¼ starting from the bregma landmark, cyan in figure. A cloud of points ranging from the left to the right lower ends of the parietal bone is recovered, green in figure, and an ellipse is fitted using least square differences, red in figure. Extracting the two sub-axes of the fitted ellipse a and b, blue in figure, and calculating the b/a ratio, produced a factor that globally characterizes the roundness of the ellipse.

A

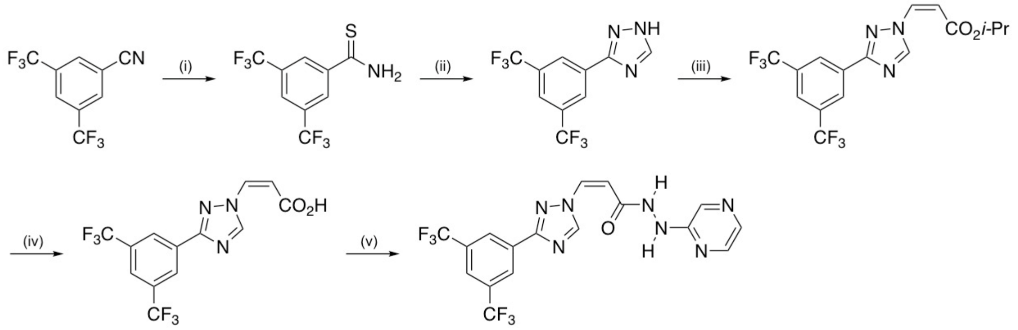

KPT-330

B

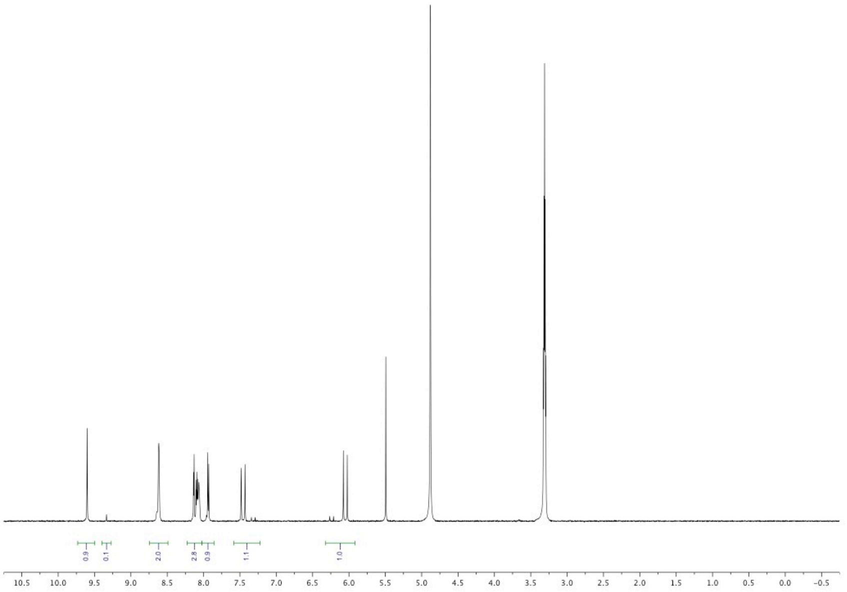

Supplementary Figure S8: Synthesis and characterization of KPT-330 (A) Synthesis intermediates, reagents and conditions (i) [20.9 mmol scale] $NaSH\cdot H_2O$, $MgCl_2$, DMF, RT, 2 h (95%); (ii) [18.3 mmol scale] formic hydrazide, 150 °C, 28 h (80%); (iii) [7.11 mmol scale] isopropyl (*Z*)-3-iodoacrylate, DABCO, DMF, RT, 2.5 h (40%); (iv) [2.54 mmol scale] KOH, H_2O/CH_3CN (1:1), RT, 26 h (quant.); (v) [1.04 mmol scale] 2-hydrazinylpyrazine, *i*-Pr_2NEt, T3P, EtOAc, 0 °C → 10 °C, 1.25 h (85%). (B) ^1H NMR spectrum of the final product (200 MHz, methanol-d_4), ~10:1 mixture of rotamers.

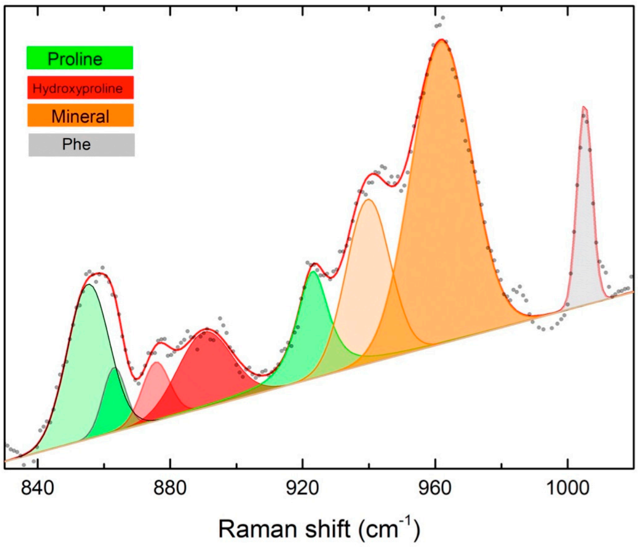

Supplementary Figure S9: Quantification of suture components. Mixed Gaussian/Lorentzian functions were used to fit the experimental Raman spectra (dotted line) to eight distinct peaks (red line). The molecular origin of each peak is indicated by color.

Supplementary Table S2. Raman spectroscopic bands assignments for bone mineral and matrix components within (830-1020 cm^{-1})

Raman shift (cm^{-1})	Assignment & Component
855	*ν(C-C),* may include *δ(C-C-H)* contribution from tyrosine, collagen proline
863	Mostly collagen proline
923	*ν(C-C)*, shoulder mostly collagen proline
875	*ν(C-C)* mostly collagen hydroxyproline
890	Mostly collagen Hydroxyproline
939	Proline and protein backbone
961	ν_1-PO_4^{3-} symmetric stretch, bone mineral
1003-1005	Ring breathing mode of Phenylalaline, collagen (Phe in supplementary figure 9)

Supplementary Table S3. The ratios calculated from the fitting parameters and the corresponding metrology

Ratio	Description	Metrology
I_{961}/I_{1005}	I_{961}, I_{1005} are the heights of 961 and 1005 cm^{-1} fitted peaks	Collagen content relative to mineral
$A_{proline}/A_{hydroxyproline}$	A: area under the fitted curve<br>$A_{proline}=A_{855}+A_{863}+A_{923}$<br>$A_{hydroxyproline}=A_{875}+A_{890}$	Proline content relative to hydroxyproline
$A_{collagen}/A_{1005}$	$A_{collagen}=A_{proline}+A_{hydroxyproline}$<br>$A_{1005}$: Area under the 1005 cm^{-1} fitted peak	Proline and Hydroxyproline content relative to phenylalanine
$A_{mineral}/A_{collagen}$	A: area under the fitted curve<br>$A_{mineral}=A_{961}$<br>$A_{collagen}=A_{proline}+A_{hydroxyproline}$	Mineral to matrix ratio
$1/W_{961}$	W_{961}: Full width at half maximum of ν_1	Mineral crystallinity
